# Z-scheme BiVO_4_/Ag/Ag_2_S composites with enhanced photocatalytic efficiency under visible light†

**DOI:** 10.1039/d0ra05712f

**Published:** 2020-08-18

**Authors:** Yi Liu, Jiajia Chen, Jinfeng Zhang, Zhongliang Tang, Haibin Li, Jian Yuan

**Affiliations:** College of Physics and Electronic Information, Huaibei Normal University Huaibei Anhui 235000 P. R. China yiliu@chnu.edu.cn; Key Laboratory of Green and Precise Synthetic Chemistry and Applications, Ministry of Education, Huaibei Normal University Huaibei Anhui 235000 P. R. China; College of Chemistry and Materials Science, Huaibei Normal University Huaibei Anhui 235000 P. R. China; Anhui Province Key Laboratory of Pollutant Sensitive Materials and Environmental Remediation, Huaibei Normal University Huaibei Anhui 235000 P. R. China

## Abstract

The Z-scheme BiVO_4_/Ag/Ag_2_S photocatalyst was fabricated *via* a two-step route. The as-prepared samples were characterized by XRD, FE-SEM, HRTEM, XPS and UV-vis diffuse reflectance spectroscopy. The results of PL and photocurrent response tests demonstrate that the ternary BiVO_4_/Ag/Ag_2_S composites had a high separation and migration efficiency of photoexcited carriers. As a result, the ternary photocatalyst exhibits enhanced photocatalytic activity for decomposing Rhodamine B (RhB) under LED light (420 nm) irradiation. The results of trapping experiments demonstrate both h^+^ and ˙OH play crucial roles in decomposing RhB molecules. Additionally, the energy band structures and density of states (DOS) of BiVO_4_ and Ag_2_S were investigated *via* the density functional theory (DFT) method. Finally, a Z-scheme electron migration mechanism of BiVO_4_ → Ag → Ag_2_S was proposed based on the experimental and calculated results.

## Abstract 2

The Z-scheme BiVO_4_/Ag/Ag_2_S photocatalyst was fabricated *via* a two-step route.

## Graphical abstract

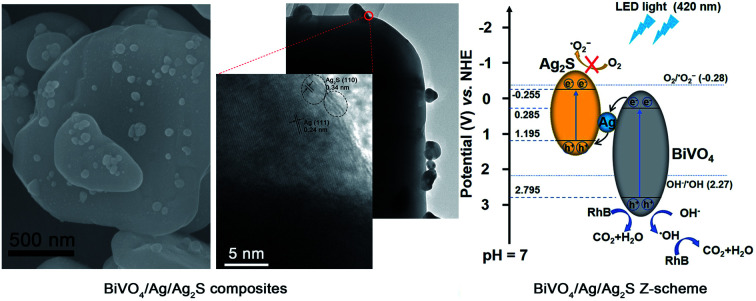

## 1 Introduction

Nowadays, photocatalysis technology is regarded as one of the best ways to alleviate energy and environmental issues. TiO_2_ is a widely known photocatalyst for water splitting and decomposing organic pollutants. However, TiO_2_ is only sensitive to UV radiation due to its large bandgap (3.2 eV). Accordingly, a maximum of 6% of solar energy can be harvested using this material, which limits its practical application.^1^ This obvious disadvantage is the main motivation for the search for new and more efficient visible-light-driven photocatalysts.

Among the developed photocatalysts, monoclinic structured BiVO_4_ has emerged as a potentially suitable visible-light-driven photocatalyst because its exceptional properties, such as narrow bandgap, good photocatalytic activity, and nontoxicity. However, the practical applications of BiVO_4_ is significantly limited owing to the weak charge-transfer rate and rapid recombination of photoinduced charge carriers.^2,3^ It has been reported that constructing semiconductor heterojunction structures could promote the photoexcited charge carrier separation, and hence improve the performance of photocatalyst. Cao *et al.* prepared Au/BiVO_4_ nanocomposites with excellent activity for water splitting owing to the improved charge separation and surface plasmon resonance of Au nanoparticles.^4^ Regmi *et al.* reported that Ag-modified BiVO_4_ sample exhibits better photocatalytic activity than BiVO_4_, which can effectively degrade bisphenol A and deactivate the *Escherichia coli*.^5^ Li *et al.* synthesized Ag_3_PO_4_/BiVO_4_ composites with type-II heterojunction, which can effectively degrade methylene blue (MB).^6^ Li *et al.* reported that the MoS_2_/BiVO_4_ composites also show excellent photoactivity for decoloring MB.^7^ Similarly, a number of studies of BiVO_4_ coupled with metal nanoparticles or other oxides were reported for the purpose of improving photocatalytic activity as well, such as Bi/BiVO_4_,^8^ Pd/BiVO_4_,^9^ CdS/BiVO_4_,^10^ g-C_3_N_4_/BiVO_4_,^11,12^ Bi_2_S_3_/BiVO_4_,^13^ WO_3_/BiVO_4_,^14^ Ag_2_O/BiVO_4_,^15^ Cu_2_O/BiVO_4_,^16^ and BiVO_4_/RGO.^17^

Ag_2_S has aroused much research interest owing to its narrow bandgap, which means that it can utilize more energy from the solar light spectrum. Therefore, Ag_2_S can act as photocatalyst or suitable sensitizer for photocatalysts under visible light irradiation. For example, Ghafoor *et al.* reported that Ag_2_S nanoparticles photosensitized TiO_2_ nanofibers display enhanced simulated solar light driven photocatalytic performance.^18^ Kumar *et al.* reported that a p–n heterojunction was fabricated by modifying NaNbO_3_ nanorods with Ag_2_S particle, which possesses excellent photoelectrochemical and photocatalytic activity.^19^ Jiang *et al.* found that compared with g-C_3_N_4_, the Ag_2_S/g-C_3_N_4_ photocatalyst exhibits improved activity for H_2_-evolution. Interestingly, Ag was formed in the process of H_2_-production. It was suggested that both Ag and Ag_2_S played a synergistic role in hydrogen production.^20^ Guan *et al.* prepared a p–n junction of Ag_2_S/BiVO_4_ with significantly improved photoelectrochemical water splitting ability.^21^ Zhao *et al.* reported that Ag_2_S/BiVO_4_ junction displays a better photocatalytic efficiency with the aid of plasmon resonance of Ag nanoparticles.^22^

However, it is known that the redox ability of photoexcited electrons and holes on reaction sites deteriorated during the charge transfer process in p–n or type-II heterojunction. As a result, the traditional semiconductor heterojunction could not exhibits both excellent charge-separation efficiency and powerful redox ability simultaneously.^23^ To overcome this drawback, the Z-scheme photocatalytic system has been developed. It was believed that the Z-scheme photocatalytic system not only promotes the separation of the photo-induced carriers, but also can optimizes carriers redox ability.^24–26^ For example, Tada *et al.* reported a Z-scheme CdS–Au–TiO_2_ composites with high photocatalytic activity, which exhibit an electron migration of TiO_2_ → Au → CdS.^27^ This unique electron migration is different with previously reported CdS/TiO_2_,^28^ which was attributed to the Au nanoparticles acting as the electron mediator. Yin *et al.* reported that C_3_N_4_/Cu_2_O photocatalyst with a p–n heterojunction can be changed to a Z-scheme system C_3_N_4_/Pd/Cu_2_O by inserting metal Pd into the C_3_N_4_/Cu_2_O interface, which has enhanced photocatalytic activity and stability.^29^ The inserted Pd nanocubes in the stack structure and the unique design are the critical roles in the formation of Z-scheme system. Deng *et al.* also synthesized a Z-scheme system BiVO_4_/Ag/Cu_2_O by deposing metallic Ag to the p–n heterojunction BiVO_4_/Cu_2_O.^30^ Lin *et al.* reported that a Z-scheme electron migration was confirmed in Ag_3_PO_4_/Ag/Bi_2_MoO_6_ composites, which is different with typical Ag_3_PO_4_/Bi_2_MoO_6_ type-II heterojunction.^31,32^ Li *et al.* found that the Ag/Ag_3_PO_4_/WO_3_ nanocomposites show better photocatalytic performance than traditional Ag_3_PO_4_/WO_3_ heterojunction, which can be attributed to the formation of Z-scheme structure.^33^ Zhao *et al.* prepared Z-scheme g-C_3_N_4_/Au/P25 composites with excellent photoactivity by introducing metal Au to the g-C_3_N_4_/P25 heterojunction.^34^ Liu *et al.* fabricated Z-scheme Cu_2_O/Au@CeO_2_ composites with strong redox ability by embedding Au nanoparticles in the yolk–shell Cu_2_O@CeO_2_ structure, which can effectively oxidize amines into imines.^35^ Bao *et al.* prepared efficient Z-scheme Cu_2_O/Cu/g-C_3_N_4_ photocatalyst for decomposing phenol *via* reduction route, in which partial metal Cu was preserved as a bridge for the transfer of photoexcited charge.^36^ Shen *et al.* reported that when reduced graphene oxide was introduced to a Cu_2_O/Fe_2_O_3_ type-II junction, the electron migration of Fe_2_O_3_ → RGO → Cu_2_O was confirmed in the Z-scheme RGO-Cu_2_O/Fe_2_O_3_ composites.^37^ Similarly, some Z-scheme photocatalysts were prepared by introducing Ag or Au to a type-II heterojunction, such as Ag_2_CO_3_/Ag/AgBr,^38^ g-C_3_N_4_/Ag/Ag_3_VO_4_ ^39^ and Au/TiO_2_-gC_3_N_4_.^40^ Therefore, it was possible that a Z-scheme photocatalytic system could be fabricated by properly adding electron mediator to a p–n or type-II heterojunction.

Herein, the Z-scheme system BiVO_4_/Ag/Ag_2_S photocatalyst was prepared *via* two-step route in this work. The photocatalytic activity of the synthesized Z-scheme BiVO_4_/Ag/Ag_2_S photocatalyst was investigated in this study. Additionally, the possible mechanism of the improved photocatalytic performance of BiVO_4_/Ag/Ag_2_S was discussed.

## 2 Experimental

### 2.1 Synthesis of photocatalysts

The BiVO_4_ sample was obtained *via* a previously reported route.^41^ 0.3 g BiVO_4_ and 0.1 mmol AgNO_3_ were added into 50 mL of distilled (DI) water and stirred for 3 h. Then, the mixture was added with 5 mL of methanol and irradiated by a UV light (100 W) for 1 h. The resulting powders (BiVO_4_/Ag composites) were collected by centrifuge and washed with DI water and ethanol, then dried at 70 °C for 8 h. 0.3 g as-synthesized BiVO_4_/Ag composites was ultrasonically dispersed in 50 mL DI water. Then, AgNO_3_ was added and stirred for 1 h. Subsequently, 50 mL of Na_2_S solution was dropped into the mixture and stirred for 8 h under dark condition. Similarly, the obtained product was centrifuged, washed and dried, in which the weight ratio of BiVO_4_, Ag and Ag_2_S is 100 : 1 : 1.

### 2.2 Characterization of the as-prepared samples

X-ray diffraction (XRD) was recorded by X-ray diffractometer (Bruker D8) with Cu Kα radiation. The samples' morphologies were characterized by field emission scanning electron microscopy (JSM 6701F) and high-resolution transmission electron microscopy (HRTEM, FEI Tecnai G2 F20 S-TWIN). X-ray photoelectron spectroscopy (XPS) was collected on ESCALAB 250Xi to identify the chemical compositions and the chemical states of the sample. UV-vis diffuse reflectance spectra (DRS) of the samples were acquired using BaSO_4_ as a reference material by a PerkinElmer Lambda 950 UV-vis spectrophotometer. Steady-state and time-resolved photoluminescence spectra (PL) were performed on FLS 920 fluorescence spectrometer.

### 2.3 Photoelectrochemical measurements

The photocurrent responses of as-synthesized photocatalysts were investigated on a Zahner PP211 electrochemical workstation in a three-electrode cell. The FTO coated with photocatalysts served as the working electrode. A Pt wire and an Ag/AgCl (saturated KCl) electrode were applied as counter and reference electrode, respectively. The photocurrent measurements were carried out in 0.1 M Na_2_SO_4_ solution. A 30 W LED lamp with lighting wavelength of 420 nm was utilized as the light source. The light spectrum of the LED lamp is presented in Fig. S1.†

### 2.4 Photocatalytic test

The photocatalytic activity of the samples was evaluated by photo-decomposing RhB. The obtained photocatalysts (50 mg) were dispersed into 50 mL RhB aqueous solution (10 mg L^−1^) and stirred in a dark condition for 30 min to ensure an adsorption–desorption equilibrium before irradiation. A LED lamp (30 W, 420 nm) was used as the visible light source. During the photodegradation experiment, the suspension was under continuous magnetic stirring. 5 mL of reaction solution was taken at 30 min intervals for analysis. The RhB concentration was monitored by Lambda 950 spectroscopy at a wavelength of 553 nm.

### 2.5 Computational details

Energy band structures and DOS of BiVO_4_ and Ag_2_S were investigated by the DFT method, in which plane-wave pseudopotential with CASTEP code was adopted. The generalized gradient approximation with the Perdew–Burke–Ernzerhof was applied as the exchange and correlation terms. The cutoff energies of 450 eV were used for all calculations. Monkhorst–Pack K-points grids of 5 × 5 × 2 and 6 × 3 × 3 were applied for BiVO_4_ and Ag_2_S, respectively.

## 3 Results and discussion

**Fig. 1:**
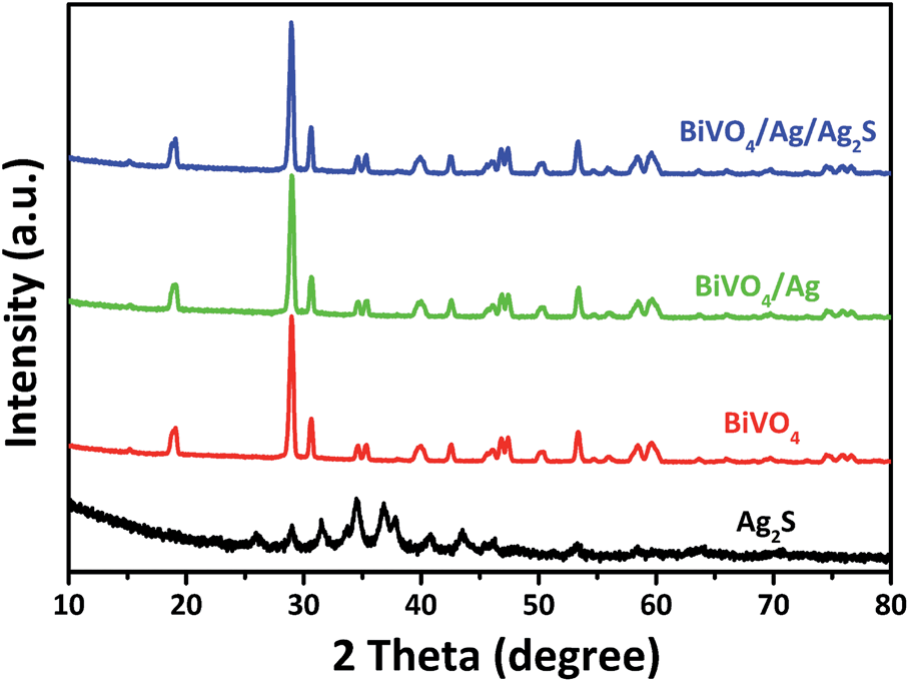
XRD patterns of the as-obtained Ag_2_S, BiVO_4_, BiVO_4_/Ag and BiVO_4_/Ag/Ag_2_S samples.

Fig. 1 presents the XRD patterns of the as-synthesized BiVO_4_, Ag_2_S, BiVO_4_/Ag and BiVO_4_/Ag/Ag_2_S samples. For the BiVO_4_ and Ag_2_S samples, all the peaks could be assigned to BiVO_4_ (JCPDS 14-0668) and Ag_2_S (JCPDS 14-0072) respectively. The XRD patterns of BiVO_4_, BiVO_4_/Ag and BiVO_4_/Ag/Ag_2_S samples were similar. No diffraction peaks from Ag are observed in the BiVO_4_/Ag sample. Similar phenomenon was observed in the XRD pattern of BiVO_4_/Ag/Ag_2_S sample and Ag_2_S was not detected as well. These results can be ascribed to the high crystallinity of BiVO_4_ powders and low contents of Ag and Ag_2_S. To further verify the existence of Ag and Ag_2_S, the as-obtained photocatalysts were further investigated by SEM, HRTEM and XPS.

**Fig. 2:**
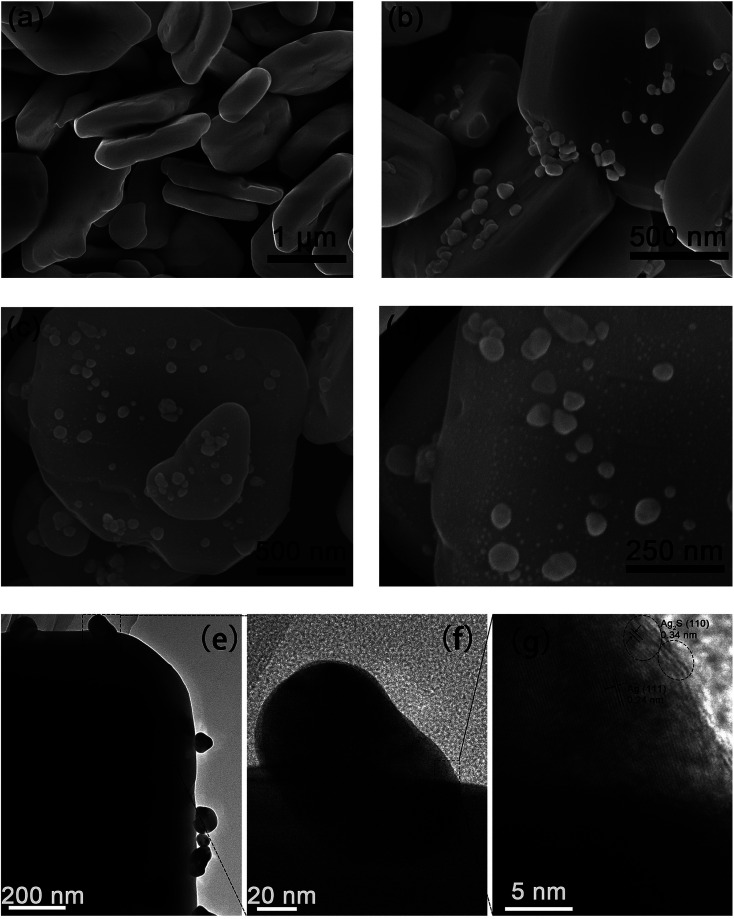
FESEM images of (a) BiVO_4_, (b) BiVO_4_/Ag and (c and d) BiVO_4_/Ag/Ag_2_S, (e and f) TEM and (g) HRTEM images of BiVO_4_/Ag/Ag_2_S composites.

Fig. 2 shows the SEM, TEM and HRTEM images of the as-obtained products. As shown in Fig. 2a, plate-like BiVO_4_ powders were obtained, which have a thickness of *ca.* 350 nm. Furthermore, the particles surface is rather smooth and clean. Fig. 2b shows the SEM image of the BiVO_4_/Ag composites. Clearly, Ag nanoparticles with diameter of 50–100 nm are attached on the surface of BiVO_4_. It was suggested that metal Ag was deposited on the plate-like BiVO_4_ particles. Fig. 2c and d present the SEM images of BiVO_4_/Ag/Ag_2_S composites with different magnification. In contrast to Fig. 2b, fine nanoparticles with a size of *ca*. 5 nm are observed on the BiVO_4_ particles, which could be referred as Ag_2_S nanoparticles formed on BiVO_4_/Ag composites. In addition, the TEM and HRTEM images of the BiVO_4_/Ag/Ag_2_S composites were recorded to further reveal its composition (Fig. 2e–g). In Fig. 2g, the lattice spacing of 0.24 nm and 0.34 nm are corresponded to the crystal plane of Ag (111) and Ag_2_S (110), respectively. This result indicates that some Ag_2_S nanoparticles were formed on the Ag particles.

**Fig. 3:**
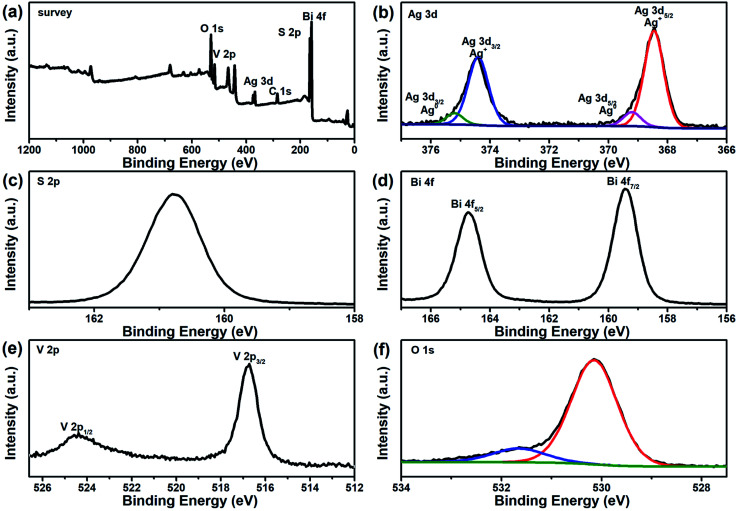
XPS spectra of BiVO_4_/Ag/Ag_2_S composites: (a) survey, (b) Ag 3d, (c) S 2p, (d) Bi 4f, (e) V 2p and (f) O 1s.

X-ray photoelectron spectroscopy (XPS) provided further evidence as to the existence of Ag and Ag_2_S in the ternary BiVO_4_/Ag/Ag_2_S composites. Ag, S, Bi, V and O elements were detected by the XPS survey spectrum of the ternary composites, as presented in Fig. 3a. From high resolution XPS analysis as depicted in Fig. 3b, two strong peaks at 373.5 and 374.2 eV were observed, which are indexed to Ag 3d_5/2_ and Ag 3d_3/2_, respectively. These two peaks could be fitted by four bands. Two peaks at 368.5 and 374.5 eV correspond to Ag^0^ 3d_5/2_ and 3d_3/2_ respectively, which demonstrated the presence of metal Ag. Another two peaks at 368.0 and 374.0 eV correspond to Ag^+^ 3d_5/2_ and 3d_3/2_ respectively. Furthermore, the peak located at 160.8 eV can be assigned to S 2p binding energy for Ag_2_S (Fig. 3c). It demonstrates that both Ag and Ag_2_S were contained in the as-prepared BiVO_4_/Ag/Ag_2_S sample. The Fig. 3d exhibits two peaks at 159.2 and 164.5 eV, which are ascribed to Bi 4f_7/2_ and Bi 4f_5/2_ of Bi^3+^ in the BiVO_4_, respectively. Two peaks at 516.9 and 524.3 eV (Fig. 3e) are attributed to V 2p_3/2_ and V 2p_1/2_ of V^5+^ in the BiVO_4_, respectively. In Fig. 3f, the O 1s binding energy was observed at about 529.9 eV. The other O 1s peak can be attributed to –OH group or chemisorbed water molecule on BiVO_4_/Ag/Ag_2_S composites surface. Basing on the XPS result, the SEM and HRTEM images, it was suggested that the ternary BiVO_4_/Ag/Ag_2_S photocatalyst was successfully fabricated.

**Fig. 4:**
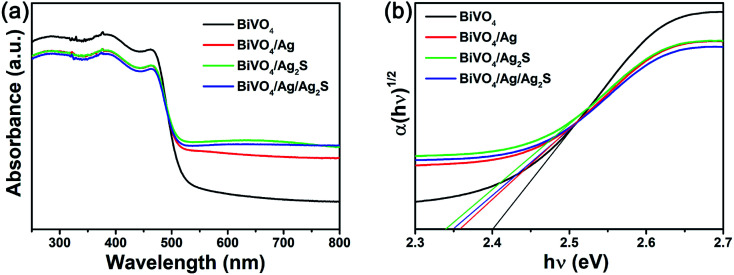
(a) UV-vis DRS absorption and (b) the plot of (*αhν*)^1/2^*vs. hν* of the as-obtained BiVO_4_, BiVO_4_/Ag, BiVO_4_/Ag_2_S and BiVO_4_/Ag/Ag_2_S photocatalysts.

Fig. 4a depicts the optical property of the as-prepared samples. It can be found that all BiVO_4_ based samples showed a strong absorption band in the 420–530 nm wavelength range, which suggested that they can absorb considerable amounts of visible light in this range. Therefore, the as-prepared BiVO_4_ based samples could act as visible-light-driven photocatalysts. Additionally, the bandgap of photocatalyst can be calculated by the following equation:^42^(*αhν*)^*n*^ = *A*(*hν* − *E*_g_)where *α*, *h*, *ν*, *E*_g_, and *A* are the absorption coefficient, Planck's constant, the light frequency, the bandgap, and a constant, respectively. *n* = 1/2 is for an indirect transmission; *n* = 2 is for a direct transmission. By plotting (*αhν*)^1/2^*vs. hν* (Fig. 4b), the bandgaps of the BiVO_4_, BiVO_4_/Ag, BiVO_4_/Ag_2_S and BiVO_4_/Ag/Ag_2_S photocatalysts are evaluated to be 2.40, 2.36, 2.34 and 2.35 eV, respectively. It suggests that the bandgap width of BiVO_4_ was slightly decreased by co-depositing Ag and Ag_2_S nanoparticles.

**Fig. 5:**
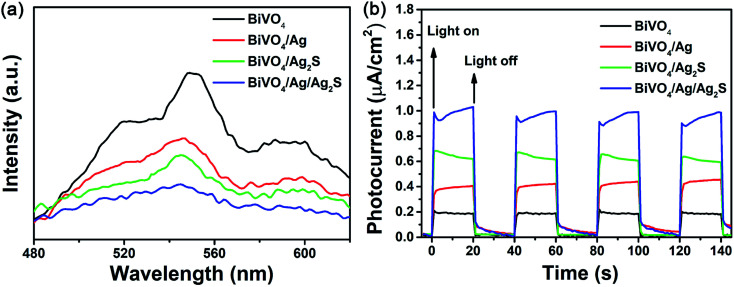
(a) PL spectrum and (b) photocurrent responses of the as-obtained BiVO_4_, BiVO_4_/Ag, BiVO_4_/Ag_2_S and BiVO_4_/Ag/Ag_2_S photocatalysts.

Fig. 5a displays the PL spectra of BiVO_4_, BiVO_4_/Ag, BiVO_4_/Ag_2_S and BiVO_4_/Ag/Ag_2_S samples. It is widely accepted that high PL intensity suggests rapid recombination of excited pairs, and results in poor photocatalytic activity. The PL peak located at about 545 nm was recorded for all samples, which was result from the photogenerated carrier recombination in BiVO_4_ particles. Compared with BiVO_4_, both the BiVO_4_/Ag and BiVO_4_/Ag_2_S display lower PL intensity, indicating that the recombination of carriers could be suppressed by modifying with Ag or Ag_2_S nanoparticles. The ternary BiVO_4_/Ag/Ag_2_S sample shows the lowest PL intensity, which implying that it has the lowest carrier recombination rate. This is also confirmed by the time-resolved PL spectra (Fig. S2 and Table S1†). It is suggested that the synergistic effect of the Ag and Ag_2_S nanoparticles play a key role to promote the separation of photoexcited carriers in the BiVO_4_/Ag/Ag_2_S composites.

To further understand the separation and migration efficiency of photoinduced electron–hole pairs, the photocurrent responses under dark and LED light irradiation were measured. As shown in Fig. 5b, the photocurrents of the prepared samples were stable and reversible at light-on and light-off. It is well known that larger photocurrent indicated a higher separation efficiency of photoexcited carriers. In comparison with BiVO_4_, BiVO_4_/Ag and BiVO_4_/Ag_2_S, BiVO_4_/Ag/Ag_2_S composites exhibited the highest photocurrent response density, about 4.5, 2.2 and 1.5 times that of BiVO_4_, BiVO_4_/Ag and BiVO_4_/Ag_2_S, respectively. It indicates that the BiVO_4_/Ag/Ag_2_S composites exhibit efficient charge separation, which is in agreement with the PL spectroscopy results.

**Fig. 6:**
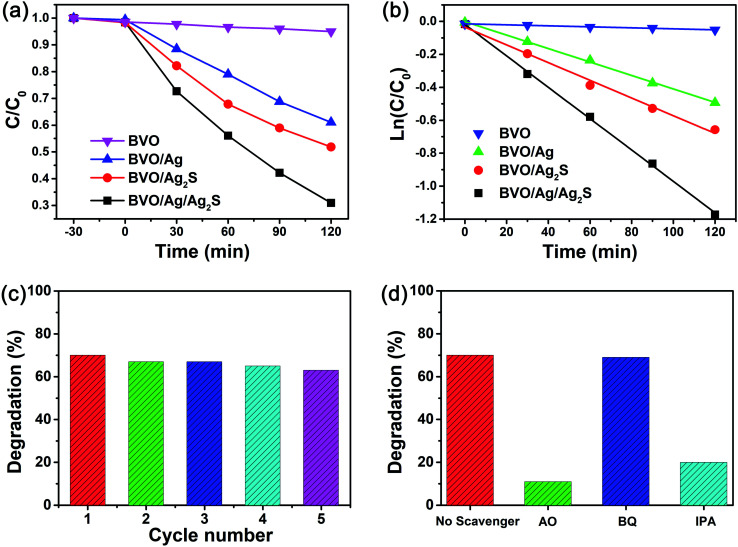
(a) Photocatalytic activity and (b) kinetic linear simulation curves of the BiVO_4_, BiVO_4_/Ag, BiVO_4_/Ag_2_S and BiVO_4_/Ag/Ag_2_S samples; (c) the reusability of the BiVO_4_/Ag/Ag_2_S composites; (d) photodegradation efficiency of RhB over the BiVO_4_/Ag/Ag_2_S samples by addition of AO, BQ and IPA.

Fig. 6a depicts the photoactivity of the as-synthesized catalysts, which was estimated by degrading RhB. When the pure BiVO_4_ sample was used as photocatalyst, only about 5% of RhB was degraded after 120 min irradiation. For the binary catalysts of BiVO_4_/Ag and BiVO_4_/Ag_2_S, about 39% and 42% of RhB were degraded, respectively. It indicates that the photocatalytic activity of BiVO_4_ could be simply enhanced by decorating with Ag or Ag_2_S nanoparticles. The BiVO_4_/Ag/Ag_2_S photocatalyst shows excellent photocatalytic performance compared with other as-prepared samples. About 69% of RhB were degraded by the ternary composites. To directly show the improved photocatalytic activity of BiVO_4_/Ag/Ag_2_S sample, the degradation kinetics for the removal of RhB were calculated by the pseudo-first-order reaction:ln *C*/*C*_0_ = −*k* × *t*where *C*_0_ and *C* are the concentration of RhB solution after adsorption and at the irradiation time *t*, respectively, *k* is the apparent reaction rate constant. As illustrated in Fig. 6b, the rate constants corresponding to BiVO_4_, BiVO_4_/Ag, BiVO_4_/Ag_2_S and BiVO_4_/Ag/Ag_2_S samples were estimated to be 0.0003, 0.0041, 0.0054 and 0.0095 min^−1^, respectively. The BiVO_4_/Ag/Ag_2_S composites show the highest *k* value, which is about 1.76, 2.32, 31.7 times higher than that of BiVO_4_/Ag_2_S, BiVO_4_/Ag and BiVO_4_, respectively. Obviously, the BiVO_4_/Ag/Ag_2_S composites shows enhanced photocatalytic efficiency, which can be attributed to the lower electron–hole pair recombination rate and higher photogenerated charge separation efficiency.

The stability of photocatalyst is a crucial issue in its practice application. Fig. 6c shows the recycled experiments of the photodegradation of RhB by using the BiVO_4_/Ag/Ag_2_S composites for five cycles (120 min LED light irradiation for each cycle). It can be seen that only slight deterioration of the BiVO_4_/Ag/Ag_2_S sample was observed after five cycles, which implies the good recyclability of the prepared photocatalyst.

As is known, the superoxide radical (˙O^2−^), holes (h^+^) and hydroxyl radical (˙OH) as active species play crucial roles in photocatalytic reaction.^43,44^ In order to evaluate the main active species, the trapping experiments were carried out. The reactive species can be removed by adding corresponding scavengers into reaction solutions. The function of different reactive species could be made clear based on the change of photocatalytic performance. In this study, *p*-benzoquinone (BQ), ammonium oxalate (AO) and isopropanol (IPA) were added to act as ˙O_2_^−^, h^+^ and ˙OH scavenger, respectively. As presented in Fig. 6d, the ability of the BiVO_4_/Ag/Ag_2_S sample to remove RhB was not affected by adding BQ, which suggesting that ˙O_2_^−^ radical does not participate in the photocatalytic reaction. However, when BQ and AO were added, the photocatalytic activity of the BiVO_4_/Ag/Ag_2_S composites decreased significantly. It demonstrates that h^+^ and ˙OH play important roles in the photocatalytic process.

To clarify the mechanism of the improved photocatalytic activity of the BiVO_4_/Ag/Ag_2_S composites, the band structure of the ternary system was studied. The band edge potentials of valence band (VB) and conduction band (CB) of BiVO_4_ and Ag_2_S can be estimated according the following equations:*E*_VB_ = *χ* − *E*^e^ + 0.5*E*_g_*E*_CB_ = *E*_VB_ − *E*_g_where *E*_VB_ and *E*_CB_ are the VB and CB edge potential, respectively; *χ* and *E*_g_ are the absolute electronegativity and bandgap energy of the semiconductor, respectively; *E*^e^ is fixed at 4.5 V. The *E*_g_ of Ag_2_S is calculated to be 1.46 eV, as shown in Fig. S3,† which is consist with previous report.^45^ Thus, the *E*_VB_ and *E*_CB_ of Ag_2_S are estimated as 1.20 and −0.26 V, *versus* the normal hydrogen electrode (NHE), respectively. Similarly, the *E*_VB_ and *E*_CB_ of BiVO_4_ are 2.795 and 0.285 V, *versus* the NHE, respectively.

**Fig. 7:**
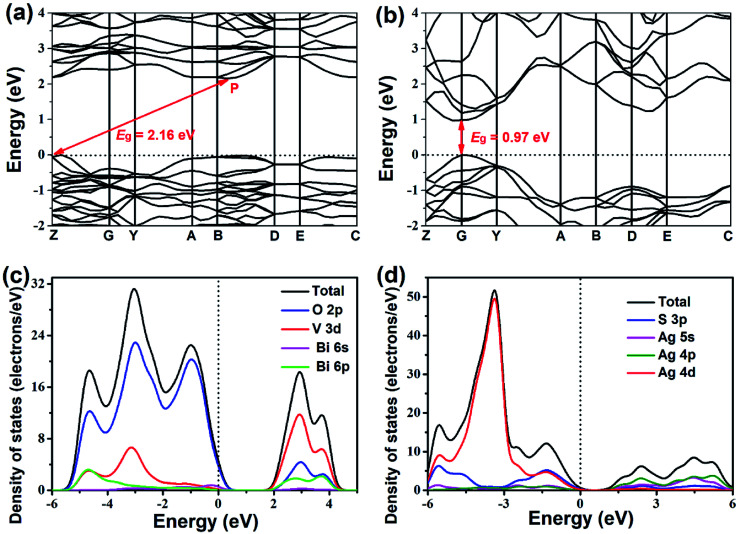
Electronic band structures of (a) BiVO_4_ and (b) Ag_2_S and density of states of (c) BiVO_4_ and (d) Ag_2_S.

Fig. 7 presents the band structures and DOS of BiVO_4_ and Ag_2_S. The valence band maximum (VBM) and conduction band minimum (CBM) are located at Z and P point, respectively (Fig. 7a), which imply that BiVO_4_ is an indirect bandgap semiconductor. Ag_2_S belongs to the direct bandgap semiconductor because the position of VBM is same as that of CBM, as demonstrated in Fig. 7b. The bandgaps of BiVO_4_ and Ag_2_S are calculated to be 2.16 eV and 0.97 eV, respectively. However, these calculated bandgaps are smaller than experimental values (2.40 eV for BiVO_4_ and 1.46 eV for Ag_2_S), which are resulted from the well-known drawback of DFT calculations.^46^ As shown in Fig. 7c, the upper VB of BiVO_4_ consists largely of O 2p, V 3d and a small amount of Bi 6s and Bi 6p states. Meanwhile, the CB of BiVO_4_ mainly consist of O 2p, V 3d and Bi 6p states. For Ag_2_S (Fig. 7d), the upper VB is mainly composed by Ag 4d states and a few of S 3p states, and the CB is mainly occupied by Ag 4p, Ag 5s and S 3p states. It is reasonable that the S 3p states result in the narrow bandgap of Ag_2_S.^47^

**Fig. 8:**
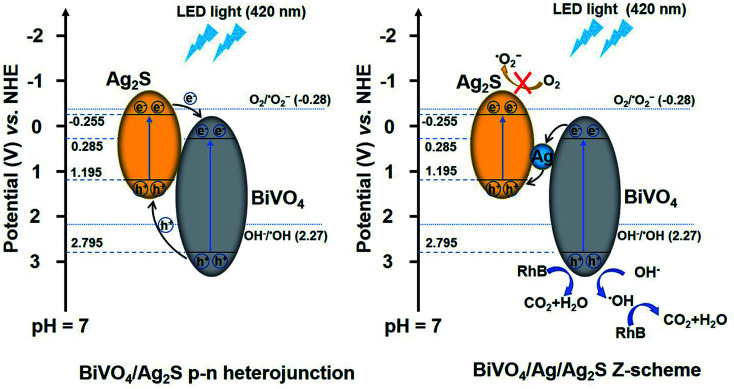
Schematic illustrating the proposed degradation mechanism of RhB over the ternary BiVO_4_/Ag/Ag_2_S composites.

Based on the above results and discussion, the mechanism of the photodecomposition of RhB with the BiVO_4_/Ag/Ag_2_S composites is proposed, as shown in Fig. 8. When the ternary BiVO_4_/Ag/Ag_2_S composites were irradiated by LED light (420 nm), electrons and holes were generated in the CB and VB of BiVO_4_ and Ag_2_S. It is important to notice that the VB potential of Ag_2_S (1.195 V *vs.* NHE) is above the ˙OH/OH^−^ potential (2.27 V *vs.* NHE at pH = 7),^48^ which demonstrating that the holes photogenerated in the VB of Ag_2_S cannot reacted with OH^−^ to produce ˙OH active species. Therefore, it is not supposed that the photoinduced holes transfer from BiVO_4_ to Ag_2_S according to the traditional p–n heterojunction. It is suggested that a Z-scheme electron migration emerges in the ternary photocatalyst. The photoexcited electrons in the CB of BiVO_4_ transferred to Ag and then recombined with the photoinduced holes in the VB of Ag_2_S, which resulting in a better charge separation. The *E*_VB_ of BiVO_4_ (3.37 V *vs.* NHE) is below the ˙OH/OH^−^ potential. Hence, the photoexcited holes in the VB of BiVO_4_ could reacted with OH^−^ to generate ˙OH.^49^ Additionally, the holes of BiVO_4_ can directly oxidized the RhB dye.^8^ Consequently, both h^+^ and ˙OH play crucial roles in decomposing RhB molecule, which is consist with the trapping experimental results.

## 4 Conclusions

The Z-scheme system BiVO_4_/Ag/Ag_2_S photocatalyst was successfully prepared *via* two-step method. The as-synthesized ternary BiVO_4_/Ag/Ag_2_S composites exhibit higher separation efficiency and reduction of carrier recombination rate, which result in enhanced photocatalytic efficiency for decomposing RhB under LED light irradiation. The photocatalytic mechanism over the ternary composites was investigated in detail. It is suggested that Z-scheme electron migration of BiVO_4_ → Ag → Ag_2_S exists in the ternary system.

## Conflicts of interest

There are no conflicts to declare.

## Supplementary Material

RA-010-D0RA05712F-s001
