# Supplementary material for: Z-scheme BiVO4/Ag/Ag2S composites with enhanced photocatalytic efficiency under visible light
Source: RSC Adv. 2020 Aug 18;10(51):30245–53. doi: 10.1039/d0ra05712f (PMC9056282; doi:10.1039/d0ra05712f)
Supplement: RA-010-D0RA05712F-s001 [file RA-010-D0RA05712F-s001.pdf]

This journal is © The Royal Society of Chemistry 2020

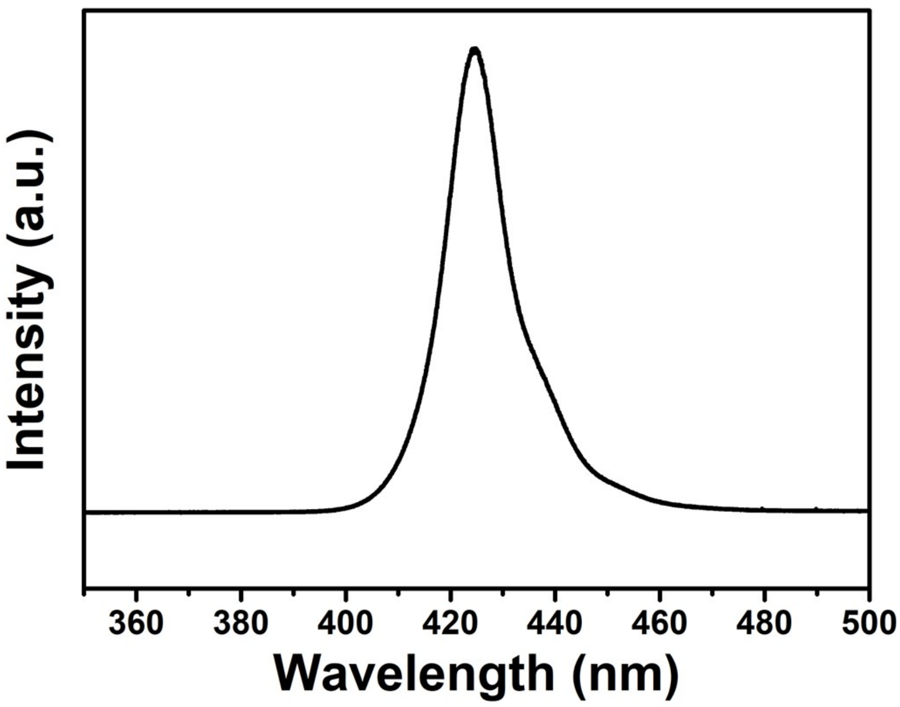

Fig. S1. The light spectrum of LED lamp (30 W).

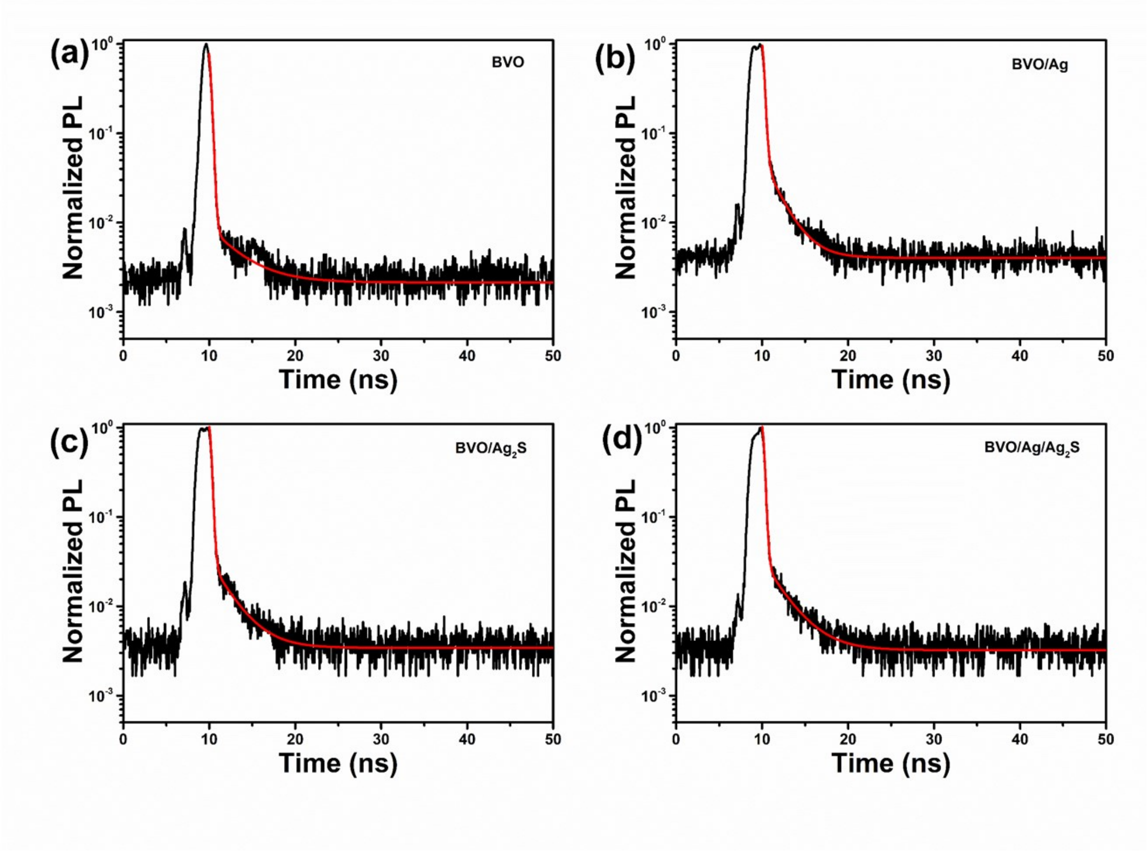

Fig. S2. Time-resolved PL decay spectra of (a) $BiVO_4$, (b) $BiVO_4$/Ag, (c) $BiVO_4$/ Ag_2S, and (d) $BiVO_4$/Ag/Ag_2S.

The recorded PL decay curves (Fig. S2) were fitted by a biexponential function. The fast decay component (τ_1) and slow component (τ_2) were calculated and listed in Table S1. τ_1 is usually arises from the nonradiative relaxation process, and τ_2 is usually attributed to the radiative process from the photoinduced carrier's recombination. The $BiVO_4$/Ag/Ag_2S sample shows the highest τ_2, suggesting a lowest recombination of photogenerated electron-hole pairs.

Table S1. Fitted parameters from time-resolved PL spectroscopy of $BiVO_4$, $BiVO_4$/Ag, $BiVO_4/Ag_2S$, and $BiVO_4/Ag/Ag_2S$ samples.

	$BiVO_4$	$BiVO_4$/Ag	$BiVO_4/Ag_2S$	$BiVO_4/Ag/Ag_2S$
τ_1 (ns)	0.16	0.19	0.16	0.17
τ_2 (ns)	1.92	1.94	2.37	2.6

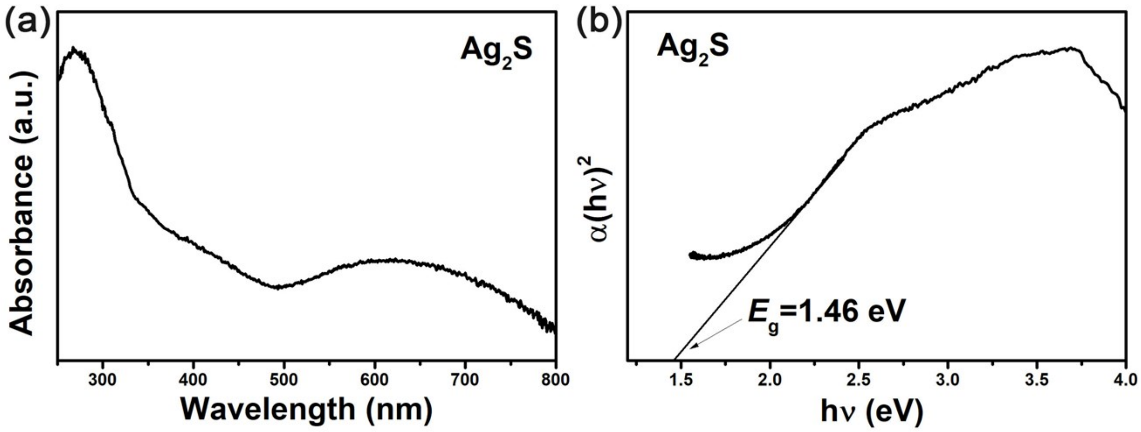

Fig. S3. (a) UV-vis DRS absorption and (b) the plot of $(\alpha h\upsilon)^2$ *vs*. $h\upsilon$ of the as-obtained Ag_2S sample.
